# Alternative Plant-Based Gluten-Free Sourdough Pastry Snack Production by Using Beetroot and Legumes: Characterization of Physical and Sensorial Attributes

**DOI:** 10.1021/acsomega.4c00515

**Published:** 2024-04-16

**Authors:** Zeynep Yolcu, Evren Demircan, Zehra Mertdinç, Elif Feyza Aydar, Beraat Özçelik

**Affiliations:** Department of Food Engineering, Faculty of Chemical and Metallurgical Engineering, Istanbul Technical University, Maslak, Istanbul 34469, Turkiye

## Abstract

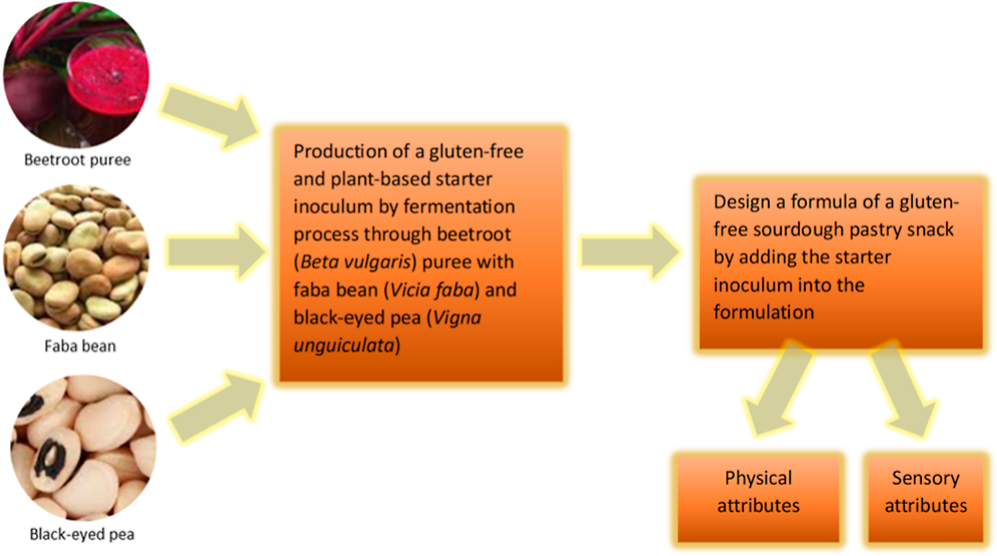

Objective of this study was to design a formula of a sourdough pastry snack by adding starter inoculum into the formulation which was obtained by the fermentation process through beetroot (*Beta vulgaris*) puree with black-eyed pea (*Vigna unguiculata*) and fava bean (*Vicia faba*). With this development process, it was aimed to review the functional impact of legumes as gluten replacement and emphasize the importance regarding physical and sensory attributes in a pastry snack product. First, a starter inoculum was developed based on modification of the shalgam fermentation process with legumes. An experimental design suggested by the response surface methodology was used to optimize its microbial properties and level of antioxidants with the factors of amounts of beetroot puree, fava bean/black-eyed pea ratio, and fermentation time. In the second part, this starter inoculum was mixed with fava bean flour to obtain a sourdough pastry snack (FBS) with improved physical and sensory attributes and compared to the wheat control sourdough (WCS) pastry snack after the baking process. According to the optimization results to produce starter inoculum with the optimum results of lactic acid bacteria 9.55 log cfu/mL, the level of antioxidant activity 91.86 μM TE/mL, and total yeast level 6.96 log cfu/mL; 75 mL of beetroot puree, 100% for fava bean, and fermentation for 24 h were obtained. Compared to WCS, FBS has approximately 16% higher hardness values. Also, a significant difference was observed for stiffness and springiness among samples. The retention of moisture was higher in the first 4 days following the storage for 8 days; the moisture content continuously decreased with the final moisture content of 12.6%. When compared with the results of textural profile analysis in terms of hardness, stiffness, and springiness, sensory results were correlated. Comparing the overall acceptability of the FBS to WCS, FBS was from moderate to higher scores, which indicated that it could be a promising alternative to chemically developed snack products and a preferred product for people suffering from celiac disease and other gluten intolerances.

## Introduction

There is an increase in consumer demand regarding functional gluten-free foods since products derived from gluten are involved in celiac disease in genetically susceptible persons. There has been increasing interest to study gluten-free grains because they make the choice foods for people suffering from celiac disease and other gluten intolerances. The typical gastrointestinal symptoms of celiac disease are abdominal pain, diarrhea, and weight loss; the silent form of celiac disease occurs often in adults, with the average worldwide prevalence estimated as high as 1:266. The current essential therapy of celiac disease is a strict adherence to a gluten-free diet.^1,2^ Celiac disease is a food-induced enteropathy in genetically susceptible individuals caused by intolerance to gluten in wheat and related proteins, such as secalins of rye, hordeins of barley, and avenins of oats. One of the distinct properties of gluten proteins that contributes to their immunogenic properties is its extreme richness in the amino acids glutamine and proline. Due to the lack of enzymes for postproline cleaving activity, the high proline content makes gluten highly resistant to proteolytic degradation within the gastrointestinal tract.^3^ Since gluten-free products available are lacking in macronutrients and phytochemicals generally, there are recent studies to design gluten-free biscuits with rice flour, plum fruit flour, and biowaste date-pit flour to enhance nutritional properties.^4^ In the research by Radhika et al.,^5^ gluten-free ingredients such as pearl millet, soya bean, finger millet, and groundnut are investigated for the development and nutritional evaluation of multigrain gluten-free cookies and pasta. In another study,^6^ while developing a gluten-free product, main focus is given to the application of arabinoxylans to substitute gluten with ohmic heating technological approach instead of conventional baking options. Although the production of gluten-free products still remains a challenge, research continues to find innovative approaches for the quality improvement such as new gluten-free ingredients, improved recipe parameters, or application of innovative baking technologies.

Considering plant-based eating, in daily consumption, diet without animal derivatives is getting an increase due to vegan diets. The demand for plant-based foods has been increasing worldwide over the years due to their potential health benefits. In overall perspective, the alternative ingredients to replace animal-based raw materials in the formulations are legumes (chickpea, or different varieties of beans), cereals (especially gluten-free corn, or rice), and pseudocereals (quinoa). It is important to emphasize that consumer preferences are changing in daily life with healthier food alternatives, at the same time sensorial properties regarding texture, flavor, and color are also important to consider while developing a product.^7^ In recent years grain legumes have been studied intensely due to their richness in protein, fibers, and other bioactive compounds, with the aim to develop functional foods with improved nutritional profile. The fortification of cereals with legume flour has been recognized as a good application to complement cereal-based food nutritional quality in bread-like products.^8^

With the increase in awareness of the relationship between health and diet, demand for new nondairy substrates for the production of fermented products has arisen.^9^ For fruits and vegetables, sugars and minerals are widely distributed, and they include the major form of soluble solids in beverages. Production of fermented beverages is performed to enhance the shelf life of the perishable ingredients used. Mainly, desired quality characteristics such as taste, mouthfeel, and texture are focused with this process to be improved. Considering major impacts of fermentation, health-promoting properties are enhanced additional to nutritional facts.^10^ Solids and compounds can be metabolized during the fermentation process by bacteria, and with these beneficial metabolites, health promoting impacts can be improved such as gastrointestinal health-related aspects.^11^

Shalgam (şalgam) beverage is a fermented soft drink with its red color and cloudy and sour profile. In traditional production, there are two stages. At stage one as dough fermentation, 30 g L^–1^ bulgur flour, 2 g L^–1^ salt, and 2 g L^–1^ sourdough (made with the incubation of baker’s yeast at 30 °C for 24 h and adequate drinkable water) are mixed and kneaded for the formation of dough. The dough is fermented in a tank at 25 °C for 3 days. After this fermentation process, 20 L of water is added into the fermented dough, blended, and extracted for 15 min. This extraction is carried out four times. The extracts obtained from the first dough fermentation are combined to perform the second fermentation step with 150 g L^–1^ chopped black carrots, 10 g L^–1^ salt, and 10 g L^–1^ sliced turnip in a 100 L of closed stainless-steel tank. If necessary, adequate water is added to fill the tank. Fermentation is carried out at 25 °C and followed daily by measuring total acidity as lactic acid and pH. Dough fermentation as the first step carried out for traditional production is not applied during the direct method as an alternative production. In that process, the tank is filled with the chopped black carrots, sliced turnip, salt, bakers’ yeast (*Saccharomyces cerevisiae*) or sourdough, and adequate water. The fermentation process occurs at ambient temperature in between 10 and 35 °C for 3 to 5 days.^12−14^

Beetroot (*Beta vulgaris*) having several varieties with different bulb colors is botanically classified as an herbaceous biennial from the Chenopodiaceae family.^15^ Beetroots with deep red coloring are the most popular for human consumption, regarding traditional cuisine, for both raw consumption and cooking. In addition to being known as fresh vegetables, or as food additives in beverages,^16,17^ it has also been found to possess as a treating and preventing ingredient of multiple diseases potentially. As a composition, beetroot contains, respectively, moisture (87.4%), total fiber (2.56%), dietary fiber (1.9%), mineral (1.4%), protein (1.35%), and fat (0.3%). It is rich in valuable active compounds such as betaine, glycine, and compounds including organic and inorganic acids, flavonoids, and phenolic acids.^18,19^

Fava bean (*Vicia faba*) has been widely used as food with its high protein content and quality of its protein.^20^ This legume is a good alternative when compared to other protein sources from animal origin because of its agronomic properties. Considering its nutritional attributes, fava beans are not commonly used in the food industry. Since fava beans are containing antinutritional factors such as raffinose family oligosaccharides or trypsin inhibitors, they might be recognized as causing problems, and for this reason, preprocessing treatments or fermentation with lactic acid bacteria (LAB) are getting important to achieve an enhanced ingredient.^21,22^ There are literature studies for fava bean in bakery products, especially in breads, however it is limited with sourdough including fava bean.^8,23^

Black-eyed pea (*Vigna unguiculata*) member of family Leguminosae, native to Asia and Africa, is a relatively inexpensive legume with high carbohydrate (65–50%) and protein (40–19%) contents. It is a warm matter whether a crop grows well in poor soils and adds nitrogen to it. As an alternative source for starch and protein production, black-eyed pea has a potential to be utilized for diverse food industrial applications.^24^

The novelty of this study lies in developing a gluten-free and plant-based sourdough pastry snack through preparation of a starter inoculum by fermentation of beetroot puree with black-eyed pea and fava bean as local legumes in an optimized level in the batter. The starter inoculum was prepared with modifying the traditional shalgam beverage production process where beetroot was used instead of black carrot/turnip and black-eyed pea/fava bean were used instead of bulgur. The prepared starter inoculum was mixed with fava bean and/or black-eyed pea flour, baked, and characterized by analyzing its physical (moisture during storage, color, and texture analysis) and sensory attributes. The fermentation process was optimized based on its microbial properties and level of antioxidants through the response surface methodology (RSM) with factors such as fermentation time, legume type (fava bean, black-eyed pea), and amount of beetroot. With this process, the objective was to demonstrate the impact of using legumes regarding physical and sensory attributes in the sourdough pastry snack product while observing their functional impact as gluten replacement.

## Results and Discussion

To gather a gluten-free design, different types of legumes such as fava bean and black-eyed pea were used to enable fermentation instead of bulgur and investigate the occurrence and growth of LAB, total yeast, and antioxidant level formation during the course of fermentation using mashed beetroot as a vegetable source. Fermented beetroot puree afterward was applied into the fava bean flour to produce a fava bean sourdough (FBS) pastry snack for further physical analysis.

RSM was used to optimize the amounts of beetroot puree (*X*_1_, 25 mL, 50 mL, and 75 mL), amount of legume (*X*_2_, 0:100, 50:50, and 100:0%), and fermentation time (*X*_3_, 16, 20, and 24 h) which were considered as three independent factors. Amount of legume type was % mixture of fava bean and black-eyed pea. The LAB, total yeast, and antioxidant level were analyzed as responses. To fit the second-order polynomial model, a Box-Behnken design was arranged. A set of 15 experiments were conducted with three replicates at the center point.

### Optimization

In this study, numerical optimization was used, and LAB, total yeast, and antioxidant level were evaluated together. According to the model, 75 mL of beetroot puree, 100% of fava bean as legume type, and 24 h fermentation time were offered to obtain LAB as 9.55 log cfu/mL, total yeast as 6.96 log cfu/mL, and antioxidant level as 91.86 μM TE/mL.

Table 1 shows the main responses for the increase of the LAB, total yeast, and antioxidant level, and this indicates that the model obtained with optimization was experimentally successful. There is not any tendency to decrease after the central point with the increase of these independent factors.

**Table 1:** Comparison of Optimized Factors with the Estimated Values from the Model

response	estimated value	average experimental result^a^	difference	*p*-value
lactic acid bacteria (log cfu/mL)	9.55	9.54 ± 0.08	0.01	0.859
total yeast (log cfu/mL)	6.96	6.95 ± 0.09	0.01	0.808
antioxidant level (μM TE/mL)	91.86	91.38 ± 1.44	0.48	0.616

a Mean ± standard deviation; *p* < 0.05 was considered statistically significant. No statistically significant difference (*p* > 0.05) was determined between the responses obtained from the optimization test in Table 1.

### Evaluation of Experimental Design

The experimental and the predicted values for the three response variables, namely, the LAB, total yeast, and antioxidant activity, are given in Table 2. As the independent factor, fava bean and black-eyed pea were used in ratios 0, 50, and 100% which means that they were added into the fermentation process in the ratios of 0:100, 50:50, and 100:0 (w/w). The level of the LAB was increased with the increase of fava bean until attained a maximum of 9.55 log cfu/mL at a fava bean percentage of 100%, with 75 mL of beetroot puree and 24 h fermentation time.

**Table 2:** Box Behnken Design for Fermented Mashed Beetroot Puree with Three Factors and Three Central Points

	coded variables^a^			uncoded variables			lactic acid bacteria (log cfu/mL)		total yeast (log cfu/mL)		antioxidant level (μM TE/mL)	
run	*X*_1_	*X*_2_	*X*_3_	*X*_1_	*X*_2_	*X*_3_	experimental^b^	predicted	experimental^b^	predicted	experimental^b^	predicted
1	0	0	0	50	50	20	5.94 ± 0.04	5.82	4.07 ± 0.05	4.05	76.96 ± 0.25	75.40
2	0	–1	1	50	0	24	6.08 ± 0.08	6.45	4.31 ± 0.05	4.60	80.59 ± 0.54	82.60
3	1	0	1	75	50	24	7.97 ± 0.05	7.79	5.86 ± 0.05	5.96	86.70 ± 0.21	83.46
4	1	0	–1	75	50	16	5.68 ± 0.17	5.93	3.26 ± 0.27	3.82	60.99 ± 4.00	61.29
5	–1	1	0	25	100	20	8.09 ± 0.05	8.25	3.58 ± 0.02	3.99	75.90 ± 0.15	74.66
6	0	1	1	50	100	24	8.54 ± 0.05	8.60	5.00 ± 0.09	5.12	81.54 ± 0.15	83.09
7	1	–1	0	75	0	20	6.03 ± 0.06	5.87	4.28 ± 0.04	3.88	84.41 ± 0.80	85.65
8	–1	0	–1	25	50	16	6.74 ± 0.13	6.92	4.78 ± 0.04	4.69	57.71 ± 2.00	60.95
9	–1	0	1	25	50	24	6.84 ± 0.08	6.58	5.01 ± 0.09	4.47	57.15 ± 1.68	56.84
10	–1	–1	0	25	0	20	5.69 ± 0.12	5.60	4.47 ± 0.30	4.71	73.02 ± 3.66	71.42
11	0	–1	–1	50	0	16	5.48 ± 0.33	5.41	3.78 ± 0.34	3.68	73.12 ± 4.99	71.57
12	0	1	–1	50	100	16	8.73 ± 0.13	8.36	4.51 ± 0.42	4.24	78.07 ± 2.73	76.06
13	0	0	0	50	50	20	5.66 ± 0.33	5.76	4.11 ± 0.09	4.11	74.61 ± 2.81	75.40
14	1	1	0	75	100	20	8.11 ± 0.05	8.19	5.92 ± 0.10	5.69	85.69 ± 2.63	87.39
15	0	0	0	50	50	20	5.78 ± 0.16	5.80	4.03 ± 0.12	4.05	74.63 ± 2.31	75.40

a *X*_1_: amount of beetroot puree (mL), *X*_2_: amount of fava bean as legume type (%), and *X*_3_: fermentation time (h).

b Mean value ± standard deviation.

**Figure 1:**
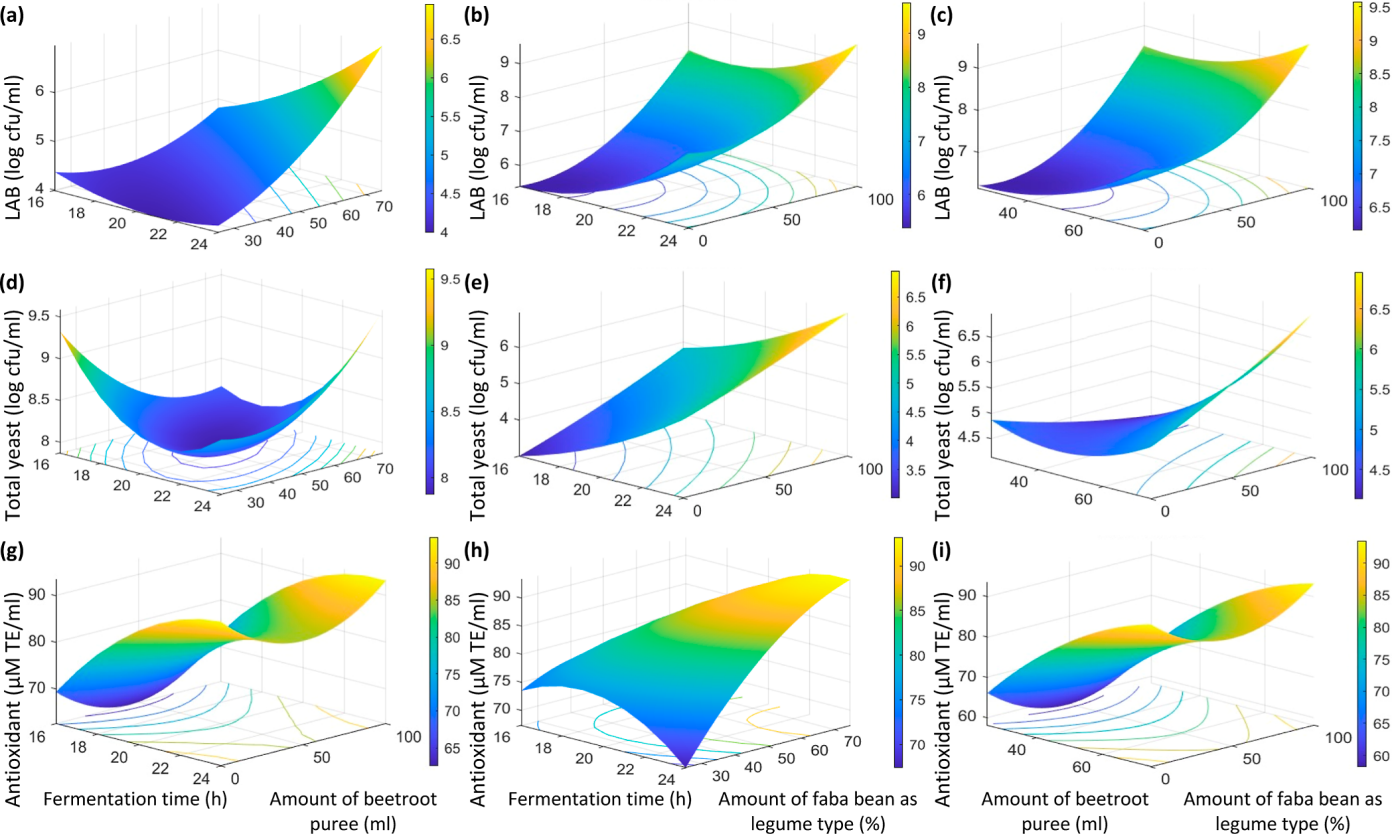
Response surface plots showing the effect of the amount of beetroot puree, fermentation time, and amount of fava bean as legume type for LAB (a–c), for total yeast (d–f), and for antioxidant (g–i).

The effect of *X*_1_, *X*_2_, and *X*_3_ on the LAB is represented in Figure 1a–c. When comparing effects of both fava bean and black-eyed pea percentage, LAB of fermented mashed beetroot juice were between 5.48 and 8.73 log cfu/mL, respectively (Table 1). In alignment with previous studies regarding results of end fermentation for shalgam beverage production, there were reported similar growth patterns for LAB using black carrot as from 7.47 to 9.01 log cfu/mL by Tanguler and Erten and from 7.66 to 7.95 log cfu/mL by Utus.^12,25^

The analysis of variance (ANOVA) for the regression parameters of the response surface model (RSM) is summarized in Table 3. The results of the ANOVA showed that the obtained regression model was statistically significant (*p* ≤ 0.05) for the antioxidant level, amount of LAB, and total yeast count with the *R*^2^ values of 93.21, 78.40, and 95.28%, and adjusted *R*^2^ values of 90.90, 71.20, and 93.71%, respectively, which comply with that models were in good agreement between the experimental and predicted values. Studies have suggested that a model’s coefficient of determination (*R*^2^) should be at least 0.80 in order to be considered a good fit. In this regard, taking total yeast value into account, even the *R*^2^ and *R*^2^ adjusted values were closer to each other and closer to the referenced value, because of significant lack of fit value, the model would specify weak relationship between the response and the predictors.^57,58^ To evaluate how effectively the model describes the response, *S* values were used. *S* is a measure of the response variable’s units that indicates the deviation of the data values from the fitted values. The lower value of *S* indicates that model describes the response better. With this information, it can be said that the obtained models of antioxidant level, amount of LAB, and total yeast count with *S* values of 2.84, 0.40, and 0.29 effectively represent responses. For the term “significant”, level of 95% and for “very significant” term, level of 99% probability test for *p*-value were used. Both *X*_1_ and *X*_3_ were very significant (*p* < 0.01) in case of modeling antioxidant values where *X*_2_ was significant (*p* < 0.05). Regarding amount of LAB, *X*_1_ was not significant (*p* > 0.05); however, *X*_2_ and *X*_3_ were very significant (*p* < 0.01). For both models, all squared terms were very significant where the interactions were of different significance.

**Table 3:** ANOVA Showing the Variables as Linear, Quadratic, and Interaction Terms for Each Response^a^

source	antioxidant level	total yeast count	amount of LAB
model	0.000**	0.000**	0.000**
*X*_1_-amount of puree	0.000**	0.031*	0.374ns
*X*_2_-grain type	0.039*	0.002**	0.000**
*X*_3_-fermentation time	0.000**	0.000**	0.000**
*X*_1_*X*_2_	0.650ns	0.000**	0.344ns
*X*_1_*X*_3_	0.000**	0.000**	0.000**
*X*_2_*X*_3_	0.231ns	0.940ns	0.025*
*X*_1_^2^	0.000**	0.002**	0.000**
*X*_2_^2^	0.000**	0.496ns	0.000**
*X*_3_^2^	0.000**	0.050*	0.000**
lack of fit	0.153ns	0.001*	0.113ns
*R*^2^	93.21%	78.40%	95.28%
adj. R^2^	90.90%	71.20%	93.71%
PRESS	520.41	10.80	5.48
*S*	2.84	0.40	0.29

a *X*_1_: amount of puree, *X*_2_: grain type, and *X*_3_: fermentation time. ns: not significant (*p* > 0.05), * value is significant at level of *p* < 0.05, ** value is very significant (*p* < 0.01). *S*: standard deviation of the distance between the data values and the fitted values. PRESS: prediction error sum of squares.

When considering macronutrients in fresh beetroot, it was reported that beetroot contains carbohydrates such as starch, sucrose, glucose, and fructose around 9.96 g per 100 g.^26^ Due to the fermentation of beetroot puree with fava bean and addition of fava flour into it to design a pastry snack product, it might be expected to have an interaction between the carbohydrate content of the beetroot and the load of the LAB in the fava bean to produce the pastry snack with improved texture.

Regarding the total yeast level, it has an increase from 3.26 to 5.92 log cfu/mL in the experimental design (Figure 1d–f). The level of the total yeast was increased with the increase of fava bean until it attained a maximum of 6.96 log cfu/mL at a fava bean percentage of 100%, with 75 mL of beetroot puree and 24 h fermentation time. Total yeast count in this study is similar to the previously reported study regarding shalgam beverage as 5.05 log cfu/mL by Ekinci et al.^27^

The level of antioxidant activity (μM TE/mL) had an increased result with the increase in fava bean until it reached to a maximum value of 91.86 μM TE/mL at a fava bean percentage of 100%, with 75 mL of beetroot puree and 24 h fermentation time. To the best of our knowledge, the literature available does not provide sufficient data regarding the content of antioxidants in the fermented beetroot with fava bean. In a recent study by Sawicki and Wiczkowski, red beet roots were peeled and chopped. After being shredded, red beet roots were mixed with salt and sugar and fermented up to 14 days.^26^ Their antioxidant capacity assays were maintained against superoxide anion radicals generated from a photosensitizer when exposed to UV light under hydrophilic condition. The antioxidant capacity was reached to the highest value on day 11 as 85.60 μM TE/mL; during these days, the fermented juice was determined with antioxidant level in 10-fold when comparing to the day 0. This observation indicates that the phenomena occurring during the fermentation process, such as the softening and the microbial activity, can cause the release of a number of phytochemicals from the beetroot matrix to the liquids, which were responsible for strong antioxidant properties. There is an increase with the level distribution from 57.15 to 86.70 μM TE/mL in the experimental design (Figure 1g–i). In previous reports, ambiguous effects were obtained for the impact of the processes such as mashing, juice extraction, or fermentation of beetroot regarding antioxidant capacity. The study by Guldiken et al.^28^ indicated a decrease in the antioxidant level during processing of beetroot while it showed an increased level in the research by Ravichandran et al.^29^ Differentiating antioxidant capacity results may indicate that it is not dependent only on the presence of betalain compounds but also on other phytochemicals possessing antioxidant potential.^30^

### Physical Properties of Sourdough Pastry Snack

Texture profile analysis (TPA) provides objective measurements of texture parameters, which indicates a major factor of food acceptability. The value of hardness with the peak force of the first and second compression, stiffness, springiness, cohesiveness, gumminess, chewiness, and adhesiveness attributes were measured in the legume-included sourdough bakery product for sensory characteristics.^1^ In this study, the TPA results (hardness with the peak force of the first and second compression, stiffness, and springiness) are summarized in Table 4, for FBS pastry snack compared to the WCS pastry snack. Hardness is the maximum force required to compress a food between the molar teeth, while stiffness is the response to a force regarding the extent to resist being deformed, and springiness represents how well a product physically springs back after deformation.^31,32^ In a general approach, the hardness of the food is being expected to be higher with increased gluten levels.^33^ It is known that the use of legume flours is usually associated with a weak structure and baking quality of the dough, to a decreased elasticity of the crumb, and to an increased hardness of the loaves in the first compression.^34^ Corresponding to the peak force of the first and second compression, FBS has a value of 172 N (hardness 1) and 6 N (hardness 2), while WCS has 148 N (hardness 1) and 25 N (hardness 2). Compared to WCS, hardness 1 and hardness 2 in FBS were approximately 16% higher and four times lower. Also, significant difference was observed for stiffness and springiness among samples (*p* < 0.05). There was no significant difference with the fracture forces (*p* > 0.05). High moisture content compared to bakery products in overall and nondevelopment of gluten can contribute into this lower hardness attribute for FBS in the second compression.^35^

**Table 4:** Texture Profile and Color Analysis of FBS and WCS^a^

		FBS	WCS
Textural Profile Analysis			
stiffness (N)		131.84 ± 5.80^a^	61.05 ± 3.27^b^
hardness 1 (N)		172.06 ± 2.70^a^	147.88 ± 1.73^b^
hardness 2 (N)		5.68 ± 1.39^b^	24.64 ± 0.39^a^
springiness (cm)		0.11 ± 0.02^b^	0.31 ± 0.12^a^
fracture force (N)		0.61 ± 0.01^b^	0.82 ± 0.11^a^
Color Analysis			
crust	L*	39.98 ± 0.33^b^	41.62 ± 0.43^a^
	a*	33.36 ± 0.36^b^	36.71 ± 1.74^a^
	b*	18.90 ± 0.02^a^	15.55 ± 0.23^b^
crumb	L*	53.07 ± 0.09^a^	52.92 ± 1.06^a^
	a*	11.39 ± 0.17^b^	23.96 ± 0.71^a^
	b*	29.49 ± 0.29^a^	19.83 ± 1.08^b^

a The data are the means of three independent experiments ± standard deviations (*n* = 3). a-b values in the same row with different superscript letters differ significantly (*p* < 0.05).

Color is one of the most important factors affecting consumer preferences for bakery products. The reactions lead to color changes at different levels in crumb and crust during baking.^36^ During fermentation, a decrease in pH positively affects the browning reactions. The duration of fermentation has been reported to influence color of the bakery product through the formation of various compounds as precursors of brown pigments.^37^ The colors through the FBS and WCS surfaces (from crust into the crumb) varied significantly, as shown in Table 4 where the L* and a* values were increased and b* values were decreased. Regarding the formula with beetroot and fava bean flour ingredients, this could be attributed by the degree of Maillard reactions and caramelization.^37^L* values of the FBS increased from the crust into the crumb, while a* values decreased. Top color of the FBS as crust with L* = 39 appeared to be darker than the crumb color with L* = 51.

**Figure 2:**
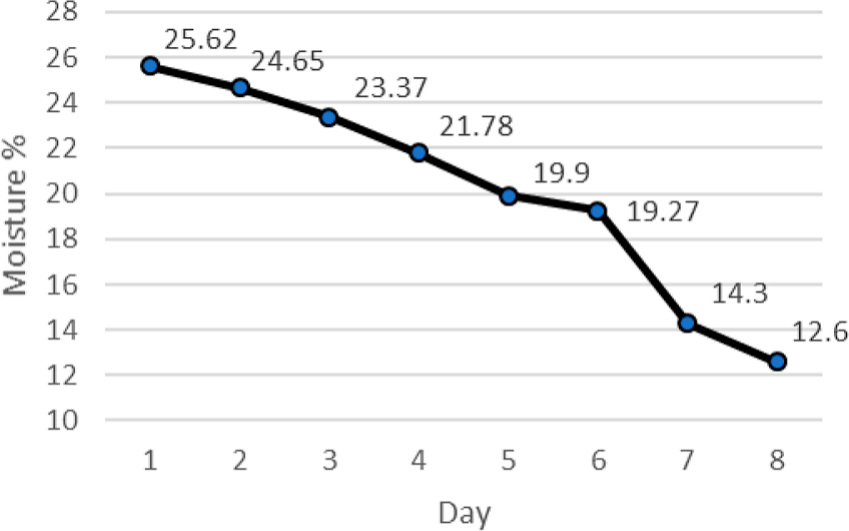
Moisture loss during the shelf life of FBS.

For bakery products, moisture retention during shelf life is important considering also high initial moisture level. One of the main results regarding the moisture decrease is the retrogradation process. As a second aspect, external polysaccharide production of LAB is thought to have a major impact on the moisture retention.^38^ As a result of fermentation, the conversion from starch to simple sugars is expected to cause moisture retention.^39^ The moisture content was 25.62% on the first day, and the retention of moisture was higher in the first 4 days when compared to the latter and as a final moisture content with 12.6% (Figure 2). Following the FBS storage for 8 days, the moisture content continuously decreased due to the moisture exchange between crust and the surrounding environment as well as water transfer at a molecular level from the protein components to starch, favoring starch retrogradation and decreasing the amount of free water in FBS.^40^

### Sensory Profile

**Figure 3:**
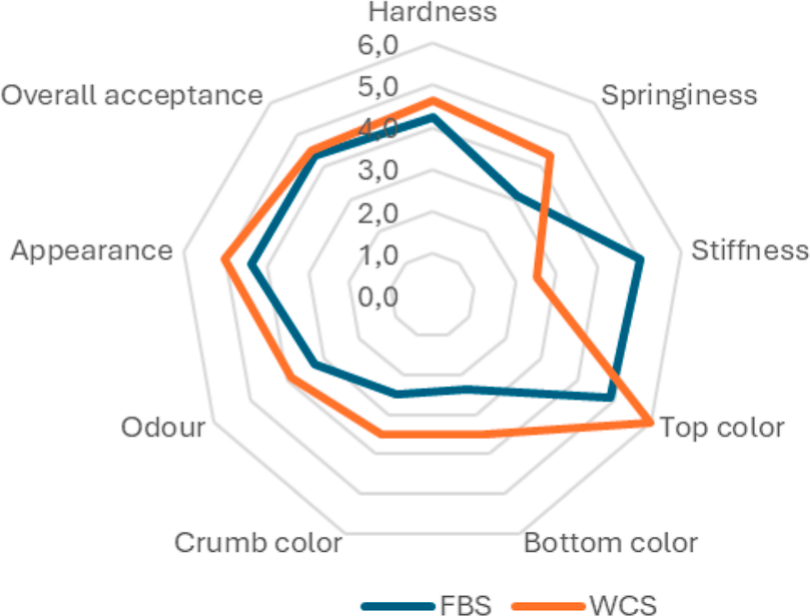
Sensory characteristics of FBS and WCS (seven-point scale).

Sensory characteristics of FBS and WCS were compared in a seven-point scale hedonic test. Figure 3 shows that FBS has lower scores in terms of appearance on the hedonic scale. Hardness and stiffness character were detected at a moderate level, while springiness is lower compared to WCS. When compared with the results of textural profile analysis in terms of hardness, stiffness, and springiness, sensory results were correlated. Regarding the color of the samples, top color as crust of WCS is dominant with a closer to red color score on the descriptive scale which indicated being similar to beetroot color. Since the starter inoculum is a kind of the sourdough type, the odor is at a moderate level due to the fermentation process and metabolites of the microbiota in the sourdough. The overall acceptability of the FBS compared to WCS was from moderate to higher scores which was similarly indicated with high acceptability as reported for bakery products containing legumes^41^ in which gluten-free bread containing chickpea exhibited good sensory behavior. Based on the results of the study by Rizzello et al.,^42^ a good acceptability of breads made with the replacement of legume flours such as lentil, bean, and chickpea was obtained as consequence of sourdough fermentation.

## Conclusions

In the present study, the fermentation process through beetroot (*B. vulgaris*) puree with black-eyed pea (*V. unguiculata*) and fava bean (*V. faba*) was investigated to develop a starter inoculum into the gluten-free sourdough pastry snack formulation. Based on the increase in consumer demand regarding functional gluten-free bakery products, it can be concluded from this study that it is applicable to use fava bean and black-eyed pea in the production of gluten-free sourdough pastry snack with acceptable physical and sensory attributes. Fermented beetroot puree used as starter inoculum for the sourdough pastry snack had a high level of LAB 9.55 log cfu/mL, total yeast 6.96 log cfu/mL, and antioxidant level 91.86 μM TE/mL which was obtained with fava bean percentage of 100%, 75 mL of beetroot puree and 24 h fermentation time.

FBS was accepted as a soft texture with a moderate hardness attribute. The overall acceptability of the FBS was from moderate to higher scores, which indicated that it could be a promising alternative to chemically developed snack products and a preferred product for people suffering from celiac disease and other gluten intolerances. Due to the best of our knowledge, the literature available does not provide sufficient data regarding alternative vegetables and legumes to be used for a fermentation process to gather bakery products with sourdough properties. Thus, future studies would be necessary to investigate the potential effects of additions on the physical and sensorial attributes of sourdough-based gluten-free bakery products.

## Materials and Methods

### Materials

Beetroot (*B. vulgaris*), black-eyed pea (*V. unguiculata*), and fava bean (*V. faba*) were purchased from a local supermarket. The chemicals used for analysis were purchased from chemical suppliers (Merck, Germany and Sigma-Aldrich, Germany). All chemicals and solvents used in this work were of analytical grade.

### Production of Fermented Beetroot Puree as Starter Inoculum

**Figure 4:**
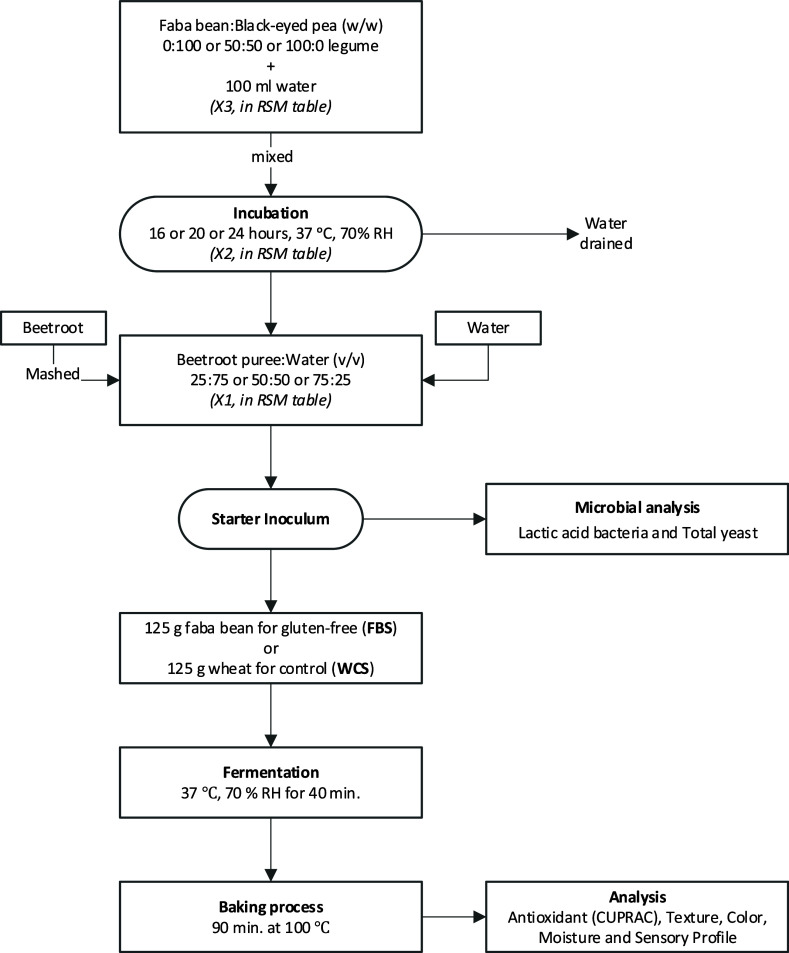
Production of fermented beetroot puree as a starter inoculum.

Traditional lactic acid fermentation was applied, with some modifications in the process. The fermentation process was conducted in two steps. In the first step, prehydration of the 50 g of legume (fava bean and/or ground black-eyed pea) with 100 mL of water was applied for 24 h at 37 °C and 70% RH. Water was drained out of the prehydrated legume, and the legume was ground. In the next step, fermentation took place, and prehydrated, drained, and ground legume was added into the beetroot puree/water with differentiating amounts (v/v: 25:75, 50:50, and 75:25). Then, sourdough pastry snack was prepared by using this fermented beetroot puree as a starter inoculum, and characterization analysis was carried out on this sourdough pastry snack (Figure 4).

Fava bean and black-eyed pea were prehydrated in water (1:2, w/v) in a covered beaker and incubated at 37 °C, 70% RH for 24 h. Then, they were drained and ground with a laboratory blender (Tefal, Turkiye). Beetroots were thoroughly washed and mashed using a food processor to prepare fermented puree (Tefal, Turkiye). Ground fava bean and/or ground black-eyed pea were mixed with mashed beetroots which was then incubated at 37 °C, 70% RH for different fermentation times that were indicated in RSM table (Table 5) and the figure (Figure 4).

**Table 5:** Factors Levels of the Independent Variables According to the Box–Behnken Design

		coded levels		
independent variables	symbol	–1	0	1
amount of beetroot puree (mL)	*X*_1_	25	50	75
amount of fava bean as legume type (%)	*X*_2_	0	50	100
fermentation time (h)	*X*_3_	16	20	24

### Experimental Design

In this study, the RSM was used to optimize the fermented beetroot puree. The amounts of beetroot puree (*X*_1_, 25, 50, and 75 mL), fava bean (*X*_2_, 0:100, 50:50, and 100:0 %), and fermentation time (*X*_3_, 16, 20, and 24 h) were considered as three independent factors, and LAB, total yeast, and antioxidant level were analyzed as responses. Box-Behnken design was arranged for fitting of a second-order polynomial model. The three factors and three replicates at the center point (runs 1, 13, and 15) led to a set of 15 experiments. The coded and uncoded values of independent variables are given in Table 5. The independent factors and their levels as well as the response variables were selected according to the results of preliminary studies.^12,43^ These studies were mainly conducted for other grain types and gluten-containing sourdough designs. It is also important to mention that some preliminary tests were performed before starting the experimental design to check the convenience of the selected levels. Amount of legume type was a % mixture of fava bean and black-eyed pea. Thus, 0, 50, and 100% of fava bean means that the ratios of fava bean/black-eyed pea in the fermentation process are 0:100, 50:50, and 100:0, respectively. The sourdough pastry snack samples were produced according to the optimization results of that experimental design.

### Production of Sourdough Pastry Snack Samples

Two snacks were prepared: FBS pastry snack and WCS pastry snack. The sourdough pastry snack production was modified based on the method from Keswet et al.^44^ Prehydrated and ground fava bean and mashed beetroots were added and incubated, as described above, at 37 °C, 70% RH for 24 h, pH up to 4.5. It was filtered by stainless-steel strainer, and approximately 80 mL of the filtrate was obtained. To produce snack batter, this filtrate as starter inoculum was mixed with fava bean flour (125 g) for FBS, followed by the main steps as kneading and fermentation at 37 °C, 70% RH for 40 min; finally baked for 90 min at 100 °C. For WCS, 125 g of wheat flour was mixed instead of fava bean flour.

### Enumeration of Lactic Acid Bacteria and Total Yeast

The microbial counts of fermented beetroot puree as a starter culture inoculum were analyzed. Numbers of LAB concentrations were determined in MRS agar as elective media (Merck, Germany). 1 mL portion of sample was mixed with sterile peptone solution and NaCl (1 g of peptone from meat/L; 8.5 g of NaCl/L) and homogenized for 1 min. Further dilutions were prepared. For LAB, the pour plate method was applied. 1 mL of dilutions were inoculated into empty plate, in duplicate. Melted MRS agar was added to the plate and swirled to mix to determine LAB. Colonies grew in and on solidified medium, under anaerobic conditions at 37 °C for 48–72 h.

Dichloran Rose Bengal chloramphenicol (DRBC) agar is a selective medium for the enumeration of molds and yeasts in foods. In the recent studies, DRBC agar was used to count yeast colonies in cereal-based fermented beverages,^47^ nondairy beverages from fermented fruit juices,^48^ and fermented pineapple juice.^49^ Since the addition of chloramphenicol serves to prevent the growth of most bacteria,^42^ DRBC agar was used for enumeration of total yeast in this study. For total yeast, the spread plate method was applied. 0.1 mL of dilutions was inoculated into plate containing solid medium (DRBC agar-Merck, Germany), in duplicate. Inoculum were spread over surface evenly. Colonies grew only on the surface of medium, under aerobic conditions at 25 °C for 96 h. At the end of incubation, the colonies were counted.^45,46^

### Determination of Total Antioxidant Capacity (TAC)

TAC was measured using the CUPRAC (Cupric Ion-Reducing Antioxidant Capacity) method according to Apak et al.^50^ A mixture of 1 mL of 0.01 M copper II chloride, 1 mL of 1 M ammonium acetate (NH_4_Ac) buffer at pH 7.0, 1 mL of 7.5 × 10^–3^ M neocuprine (Nc) solution (in 96% ethanol), and 1 mL of distilled water was prepared. Samples were grounded and mixed with 75% methanol solution, centrifuged, and supernatant phase obtained for the antioxidant analysis. 100 μL of sample solution and 4 mL of mixture were added and mixed well (total volume: 4.1 mL). This final mixture in a stoppered test tube was allowed to stand at room temperature for 30 min in dark. Then, the absorbance was measured in a spectrophotometer (Biotech, Synergy HT, USA) against a reagent blank at 450 nm. All measurements were made in triplicates, and results were mentioned as μM TE (trolox equivalent) per mL sample.

### Physical Properties

#### Texture Analysis of FBS and WCS

The textural properties of FBS and WCS were evaluated by using a TAplus texture analyzer (Lloyd Instruments, Ametek, UK). Samples were cut in size of 20 mm thick slices, performed by using a 35 mm diameter probe SMS P/36, 1-kg load cell, 50% penetration depth. The compression rate was 5 mm/s; pretest and test speed were 1.7 mm/s, and post-test speed was 10 mm/s.^51^ The parameters hardness 1, hardness 2, springiness, stiffness, and fracture force were obtained.

#### Moisture Analysis of FBS

After 3 h from baking, the round mold slices were homogenized including crust and crumb, and 5 g was weighed out. Moisture was determined by oven drying at 105 °C to constant weight.^52^ The FBS samples were wrapped in plastic bags and stored at the same room conditions (19 ± 2 °C) and kept away from direct sunlight. During storage, moisture loss was determined between days 0 and 8.

#### Color Analysis of FBS and WCS

The color of crust and crumb of FBS and WCS was determined according to Torrieri et al.^53^ Analyses were performed using a colorimeter (Konica Minolta, Chroma Meter CR 400, Japan) in the form of L*, a*, and b* (L*: lightness; a*: red; and b*: yellow), by applying a CIELAB color scale and direct reading of the reflectance of the rectangular coordinate system. This instrument was calibrated with a white standard tile before the measurements. The bread crust and crumb colors were examined separately. Crust color was measured at different positions on top of the sourdough pastry snack slices. The measurement of crumb color was carried out in the middle of each slice. Mean values were calculated of three measurements.^54^

### Sensory Profile

Sensory evaluation of FBS and WCS was carried out within 24 h after baking and assessed by eight trained panelists (consisting of seven females and one male of age groups ranging from 24 to 41 years, nonsmokers) from the Department of Food Engineering in Istanbul Technical University. A seven-point scale was used for the descriptive test (1: none, 7: too much) and for the hedonic test (1: dislike extremely, 7: like extremely). The attributes were evaluated for the descriptive properties in terms of hardness, springiness, stiffness, top color, bottom color, and crumb color and for the hedonic test in terms of odor, appearance, and overall acceptance for preference.^55,56^ The participants were informed about ingredients, and a short training has been given to panelists about attributes that will be evaluated during the test. A microbiologically safe environment could not be provided; hence, the samples were not allowed to be consumed, and the taste attribute was discarded. Since it is obligatory to grant an ethical statement as required for the sensory analysis, the participants did not consume any samples. The results were presented as sensory diagrams.

### Statistical Analysis

The results are presented as an average of triplicate measurements. The statistical difference was measured with one-way ANOVA (Minitab 16, USA). The effect of treatments was measured with Tukey’s test with significance level of *p*< 0.05.

## Ethical Statement

Although an ethical statement was required for the sensory analysis, it was not mandatory to grant an ethical statement as the participants did not consume any samples. In addition, the participants were informed to consent via the statement “I am aware that my responses are confidential, and I agree to participate in this survey”, where an affirmative reply was required to enter the sensory analysis. They were able to withdraw from the survey at any time without giving a reason.

## References

[ref1] Olojede A. O.; Sanni A. I.; Banwo K. Effect of Legume Addition on the Physiochemical and Sensorial Attributes of Sorghum-Based Sourdough Bread. LWT-Food Sci. Technol. 2020, 118, 108769–108776. 10.1016/j.lwt.2019.108769.

[ref2] Rai S.; Kaur A.; Chopra C. S. Gluten-Free Products for Celiac Susceptible People. Front. Nutr. 2018, 5, 116 10.3389/fnut.2018.00116. 30619866 PMC6304385

[ref3] Dunaevsky Y. E.; Tereshchenkova V. F.; Belozersky M. A.; Filippova I. Y.; Oppert B.; Elpidina E. N. Effective Degradation of Gluten and Its Fragments by Gluten-Specific Peptidases: A Review on Application for the Treatment of Patients with Gluten Sensitivity. Pharmaceutics 2021, 13, 1603 10.3390/pharmaceutics13101603. 34683896 PMC8541236

[ref4] Saeed S.; Ali S.; Faheem K.; Ali R.; Giuffrè A. The Impact of Innovative Plant Sources (Cordia myxa L. Fruit (Assyrian Plum) and Phoenix dactylifera L. Biowaste (Date Pit)) on the Physicochemical, Microstructural, Nutritional, and Sensorial Properties of Gluten-Free Biscuits. Foods 2022, 11 (15), 2346 10.3390/foods11152346. 35954112 PMC9368538

[ref5] Radhika R.; Virk A.; Kaur M.; Thakur P.; Chauhan D.; Rizvi Q. U. E. H.; Jan S.; Kumar K. Development and nutritional evaluation of multigrain gluten free cookies and pasta products. Curr. Res. Nutr. Food Sci. 2019, 7, 842–853. 10.12944/CRNFSJ.7.3.23.

[ref6] Bender D.; Schonlechner R. Innovative approaches towards improved gluten-free bread properties. J. Cereal. Sci. 2020, 91, 102904–102908. 10.1016/j.jcs.2019.102904.

[ref7] Fructuoso I.; Romão B.; Han H.; Raposo A.; Ariza-Montes A.; Araya-Castillo L.; Zandonadi R. P. An Overview on Nutritional Aspects of Plant-Based Beverages Used as Substitutes for Cow’s Milk. Nutrients 2021, 13, 2650 10.3390/nu13082650. 34444815 PMC8399839

[ref8] Angioloni A.; Collar C. High Legume-Wheat Matrices: An Alternative to Promote Bread Nutritional Value Meeting Dough Viscoelastic Restrictions. Eur. Food Res. Technol. 2012, 234, 273–284. 10.1007/s00217-011-1637-z.

[ref9] Yang X.; Zhou J.; Fan L.; Qin Z.; Chen Q.; Zhao L. Antioxidant Properties of a Vegetable-Fruit Beverage Fermented with Two lactobacillus plantarum Strains. Food Sci. Biotechnol. 2018, 27, 1719–1726. 10.1007/s10068-018-0411-4. 30483436 PMC6233392

[ref10] De Roos J.; De Vuyst L. Acetic Acid Bacteria in Fermented Foods and Beverages. Curr. Opin. Biotechnol. 2018, 49, 115–119. 10.1016/j.copbio.2017.08.007. 28863341

[ref11] Somanah J.; Bourdon E.; Rondeau P.; Bahorun T.; Aruoma O. I. Relationship between Fermented Papaya Preparation Supplementation, Erythrocyte Integrity and Antioxidant Status in Pre-Diabetics. Food Chem. Toxicol. 2014, 65, 12–17. 10.1016/j.fct.2013.11.050. 24316314

[ref12] Tanguler H.; Erten H. Occurrence and Growth of Lactic Acid Bacteria Species during the Fermentation of Shalgam (Salgam), a Traditional Turkish Fermented Beverage. LWT-Food Sci. Technol. 2012, 46, 36–41. 10.1016/j.lwt.2011.10.026.

[ref13] Toktas B.; Bildik F.; Ozcelik B. Effect of fermentation on anthocyanin stability and in vitro bioaccessibility during shalgam (salgam) beverage production. J. Sci. Food Agric. 2018, 98 (8), 3066–3075. 10.1002/jsfa.8806. 29194639

[ref14] Erten H.; Tanguler H.; Canbas A. A traditional Turkish lactic acid fermented beverage: shalgam (salgam). Food Rev. Int. 2008, 24, 352–359. 10.1080/87559120802089324. PMC5495728 28720958

[ref15] Chen L.; Zhu Y.; Hu Z.; Wu S.; Jin C. Beetroot as a Functional Food with Huge Health Benefits: Antioxidant, Antitumor, Physical Function, and Chronic Metabolomics Activity. Food Sci. Nutr. 2021, 9, 6406–6420. 10.1002/fsn3.2577. 34760270 PMC8565237

[ref16] Vieira Teixeira da Silva D.; dos Santos Baião D.; de Oliveira Silva F.; Alves G.; Perrone D.; Mere Del Aguila E.; M Flosi Paschoalin V. Betanin, a Natural Food Additive: Stability, Bioavailability, Antioxidant and Preservative Ability Assessments. Molecules 2019, 24, 458 10.3390/molecules24030458. 30696032 PMC6384587

[ref17] Georgiev V. G.; Weber J.; Kneschke E. M.; Denev P. N.; Bley T.; Pavlov A. I. Antioxidant Activity and Phenolic Content of Betalain Extracts from Intact Plants and Hairy Root Cultures of the Red Beetroot Beta Vulgaris Cv. Detroit Dark Red. Plant Foods Hum. Nutr. 2010, 65, 105–111. 10.1007/s11130-010-0156-6. 20195764

[ref18] Singh B.; Hathan B. S. Chemical Composition, Functional Properties, and Processing of Beetroot-a Review. Int. J. Sci. Eng. Res. 2014, 5, 679–684.

[ref19] Kujala T. S.; Loponen J. M.; Klika K. D.; Pihlaja K. Phenolics and Betacyanins in Red Beetroot (Beta Vulgaris) Root: Distribution and Effect of Cold Storage on the Content of Total Phenolics and Three Individual Compounds. J. Agric. Food Chem. 2000, 48, 5338–5342. 10.1021/jf000523q. 11087483

[ref20] Crépon K.; Marget P.; Peyronnet C.; Carrouée B.; Arese P.; Duc G. Nutritional Value of Faba Bean (Vicia faba L.) Seeds for Feed and Food. Field Crops Res. 2010, 115, 329–339. 10.1016/j.fcr.2009.09.016.

[ref21] Multari S.; Stewart D.; Russell W. R. Potential of Fava Bean as Future Protein Supply to Partially Replace Meat Intake in the Human Diet. Compr. Rev. Food Sci. Food Saf. 2015, 14, 511–522. 10.1111/1541-4337.12146.

[ref22] Youssef M. M.; Bushuk W. Breadmaking Properties of Composite Flours of Wheat and Faba Bean Protein Preparations. Cereal Chem. 1986, 63, 357–361.

[ref23] Petitot M.; Boyer L.; Minier C.; Micard V. Fortification of Pasta with Split Pea and Faba Bean Flours: Pasta Processing and Quality Evaluation. Food Res. Int. 2010, 43, 634–641. 10.1016/j.foodres.2009.07.020.

[ref24] Prinyawiwatkul W.; McWatters K. H.; Beuchat L. R.; Phillips R. D. Functional Characteristics of Cowpea (Vigna unguiculata) Flour and Starch as Affected by Soaking, Boiling, and Fungal Fermentation before Milling. Food Chem. 1997, 58, 361–372. 10.1016/S0308-8146(96)00259-2.

[ref25] Utus D. The Effect of Black Carrot (Daucus carota) Size Used on the Quality of Shalgam Production. M.Sc. Thesis, Cukurova University, Turkiye, 2008.

[ref26] Sawicki T.; Wiczkowski W. The Effects of Boiling and Fermentation on Betalain Profiles and Antioxidant Capacities of Red Beetroot Products. Food Chem. 2018, 259, 292–303. 10.1016/j.foodchem.2018.03.143. 29680057

[ref27] Ekinci F. Y.; Baser G. M.; Ozcan E.; Ustundag O. G.; Korachi M.; Sofu A.; Blumberg J. B.; Chen C. Y. O. Characterization of chemical, biological, and antiproliferative properties of fermented black carrot juice, shalgam. Eur. Food Res. Technol. 2016, 242, 1355–1368. 10.1007/s00217-016-2639-7.

[ref28] Guldiken B.; Toydemir G.; Nur Memis K.; Okur S.; Boyacioglu D.; Capanoglu E. Home-Processed Red Beetroot (Beta vulgaris L.) Products: Changes in Antioxidant Properties and Bioaccessibility. Int. J. Mol. Sci. 2016, 17, 858 10.3390/ijms17060858. 27258265 PMC4926392

[ref29] Ravichandran K.; Saw N. M. M. T.; Mohdaly A. A. A.; Gabr A. M. M.; Kastell A.; Riedel H.; Cai Z.; Knorr D.; Smetanska I. Impact of Processing of Red Beet on Betalain Content and Antioxidant Activity. Food Res. Int. 2013, 50, 670–675. 10.1016/j.foodres.2011.07.002.

[ref30] Chhikara N.; Kushwaha K.; Sharma P.; Gat Y.; Panghal A. Bioactive Compounds of Beetroot and Utilization in Food Processing Industry: A Critical Review. Food Chem. 2019, 272, 192–200. 10.1016/j.foodchem.2018.08.022. 30309532

[ref31] Chandra M. V.; Shamasundar B. A. Texture Profile Analysis and Functional Properties of Gelatin from the Skin of Three Species of Fresh Water Fish. Int. J. Food Prop. 2015, 18, 572–584. 10.1080/10942912.2013.845787.

[ref32] Nicole T. Z. H.; Nichelle T. S.; Elizabeth T. E.; Yuliarti O. Formulation of Functional Crackers Enriched with Fermented Soybean (Tempeh) Paste: Rheological and Microstructural Properties. Future Foods 2021, 4, 100050–100059. 10.1016/j.fufo.2021.100050.

[ref33] Kohajdova Z.; Karovicova J.; Magala M. Effect of lentil and bean flours on rheological and baking properties of wheat dough. Chem. Pap. 2013, 67, 398–407. 10.2478/s11696-012-0295-3.

[ref34] Miñarro B.; Albanell E.; Aguilar N.; Guamis B.; Capellas M. Effect of Legume Flours on Baking Characteristics of Gluten-Free Bread. J. Cereal Sci. 2012, 56, 476–481. 10.1016/j.jcs.2012.04.012.

[ref35] Artan M. Y.; Karim R.; Chern B. H.; Ariffin A. A.; Man Y. C.; Chin N. L. The Influence of Different Formulations Palm Oil-Palm Stearin-Based Shortening on the Quality of White Bread. Middle East J. Sci. Res. 2010, 5, 469–476.

[ref36] Martínez-Anaya M. A. Enzymes and Bread Flavor. J. Agric. Food Chem. 1996, 44, 2469–2480. 10.1021/jf960020d.

[ref37] Suriya M.; Rajput R.; Reddy C. K.; Haripriya S.; Bashir M. Functional and Physicochemical Characteristics of Cookies Prepared from Amorphophallus Paeoniifolius Flour. J. Food Sci. Technol. 2017, 54, 2156–2165. 10.1007/s13197-017-2656-y. 28720973 PMC5495745

[ref38] Galle S.; Arendt E. K. Exopolysaccharides from Sourdough Lactic Acid Bacteria. Crit. Rev. Food Sci. Nutr. 2014, 54, 891–901. 10.1080/10408398.2011.617474. 24499068

[ref39] Ertop M. H.; İbrahim Tuğkan S. Optimization of the Amount of Chickpea Sourdough and Dry Yeast in Wheat Bread Formulation: Evaluation of Physicochemical, Sensory and Antioxidant Properties. Food Sci. Technol. Res. 2018, 24, 45–53. 10.3136/fstr.24.45.

[ref40] Ronda F.; Roos Y. H. Staling of fresh and frozen gluten-free bread. J. Cereal. Sci. 2011, 53, 340–346. 10.1016/j.jcs.2011.02.004.

[ref41] Ndife J.; Abdulraheem L. O.; Zakari U. M. Evaluation of the Nutritional and Sensory Quality of Functional Breads Produced from Whole Wheat and Soya Bean Flour Blends. Afr. J. Food Sci. 2011, 5, 466–472.

[ref42] Rizzello C. G.; Calasso M.; Campanella D.; De Angelis M.; Gobbetti M. Use of sourdough fermentation and mixture of wheat, chickpea, lentil and bean flours for enhancing the nutritional, texture and sensory characteristics of white bread. Int. J. Food Microbiol. 2014, 180, 78–87. 10.1016/j.ijfoodmicro.2014.04.005. 24794619

[ref43] Lattanzi A.; Minervini F.; Di Cagno R.; Diviccaro A.; Antonielli L.; Cardinali G.; Cappelle S.; De Angelis M.; Gobbetti M. The Lactic Acid Bacteria and Yeast Microbiota of Eighteen Sourdoughs Used for the Manufacture of Traditional Italian Sweet Leavened Baked Goods. Int. J. Food Microbiol. 2013, 163, 71–79. 10.1016/j.ijfoodmicro.2013.02.010. 23558189

[ref44] Keswet L. M.; Ayo J. A.; Bello C. B. The Effect of Four Nigerian Wheat Flours on the Loaf Volume and Sensory Quality of Bread. Nutr. Food Sci. 2003, 33, 34–37. 10.1108/00346650310459554.

[ref45] Lonner C.; Preve-Åkesson K. Acidification Properties of Lactic Acid Bacteria in Rye Sour Doughs. Food Microbiol. 1988, 5, 43–58. 10.1016/0740-0020(88)90007-X.

[ref46] Şimsek Ö.; Çon A. H.; Tulumoglu Ş. Isolating Lactic Starter Cultures with Antimicrobial Activity for Sourdough Processes. Food Control 2006, 17, 263–270. 10.1016/j.foodcont.2004.10.011.

[ref47] Gebre T. S.; Emire S. A.; Chelliah R.; Aloo S. O.; Oh D. H. Isolation, functional activity, and safety of probiotics from Ethiopian traditional cereal-based fermented beverage, “Borde”. LWT–Food Sci. Technol. 2023, 184, 115076 10.1016/j.lwt.2023.115076.

[ref48] Randazzo W.; Corona O.; Guarcello R.; Francesca N.; Germana M. A.; Erten H.; Moschetti G.; Settanni L. Development of new non-dairy beverages from Mediterranean fruit juices fermented with water kefir microorganisms. Food Microbiol. 2016, 54, 40–51. 10.1016/j.fm.2015.10.018.

[ref49] Chanprasartsuk O.; Prakitchaiwattana C. Growth kinetics and fermentation properties of autochthonous yeasts in pineapple juice fermentation for starter culture development. Int. J. Food Microbiol. 2022, 371, 109636 10.1016/j.ijfoodmicro.2022.109636. 35447561

[ref50] Apak R.; Güçlü K.; Özyürek M.; Karademir S. E. Novel Total Antioxidant Capacity Index for Dietary Polyphenols and Vitamins C and E, Using Their Cupric Ion Reducing Capability in the Presence of Neocuproine: CUPRAC Method. J. Agric. Food Chem. 2004, 52, 7970–7981. 10.1021/jf048741x. 15612784

[ref51] Coda R.; Varis J.; Verni M.; Rizzello C. G.; Katina K. Improvement of the Protein Quality of Wheat Bread through Faba Bean Sourdough Addition. LWT-Food Sci. Technol. 2017, 82, 296–302. 10.1016/j.lwt.2017.04.062.

[ref52] Akgun N. A.; Doymaz I. Modelling of Olive Cake Thin-Layer Drying Process. J. Food Eng. 2005, 68, 455–461. 10.1016/j.jfoodeng.2004.06.023.

[ref53] Torrieri E.; Pepe O.; Ventorino V.; Masi P.; Cavella S. Effect of Sourdough at Different Concentrations on Quality and Shelf Life of Bread. LWT-Food Sci. Technol. 2014, 56, 508–516. 10.1016/j.lwt.2013.12.005.

[ref54] Phattanakulkaewmorie N.; Paseephol T.; Moongngarm A. Chemical Compositions and Physico-Chemical Properties of Malted Sorghum Flour and Characteristics of Gluten Free Bread. World Acad. Sci. Eng. Technol. 2011, 81, 454–460.

[ref55] Ogunsakin O. A.; Banwo K.; Ogunremi O. R.; Sanni A. I. Microbiological and Physicochemical Properties of Sourdough Bread from Sorghum Flour. Int. Food Res. J. 2015, 22, 2610–2618.

[ref56] Volpini-Rapina L. F.; Sokei F. R.; Conti-Silva A. C. Sensory Profile and Preference Mapping of Orange Cakes with Addition of Prebiotics Inulin and Oligofructose. LWT-Food Sci. Technol. 2012, 48, 37–42. 10.1016/j.lwt.2012.03.008.

[ref57] Koocheki A.; Taherian A. R.; Razavi S. M. A.; Bostan A. Response surface methodology for optimization of extraction yield, viscosity, hue and emulsion stability of mucilage extracted from Lepidium perfoliatum seeds. Food Hydrocolloids 2009, 23 (8), 2369–2379. 10.1016/j.foodhyd.2009.06.014.

[ref58] Srivastava U.; Saini P.; Ahmed M.; Singh A. Enhancing antioxidant activity in barnyard millet fermentation through RSM optimization. Food Humanity 2024, 2, 100254 10.1016/j.foohum.2024.100254.

